# Elevated peak width of skeletonized mean diffusivity in patients with end-stage kidney disease: evidence of diffuse white matter microstructural injury

**DOI:** 10.3389/fneur.2026.1877913

**Published:** 2026-09-18

**Authors:** Junghae Ko, Bong Soo Park, Chang Min Heo, Sihyung Park, Jiyae Yi, Ho-Joon Lee, Dong Ah Lee, Kang Min Park

**Affiliations:** 1 Department of Internal Medicine, Haeundae Paik Hospital, College of Medicine, Inje University, Busan, Republic of Korea; 2 Department of Radiology, Haeundae Paik Hospital, College of Medicine, Inje University, Busan, Republic of Korea; 3 Department of Neurology, Haeundae Paik Hospital, College of Medicine, Inje University, Busan, Republic of Korea

**Keywords:** cerebral small vessel diseases, diffusion tensor imaging, kidney failure, MRI, PSMD

## Abstract

**Background:**

Peak width of skeletonized mean diffusivity (PSMD) is a diffusion tensor imaging-based marker of diffuse white matter microstructural injury that has been associated with cerebral small vessel disease (CSVD). This study aimed to investigate whether patients with end-stage kidney disease (ESKD) exhibit altered PSMD compared with healthy controls.

**Methods:**

We prospectively enrolled 38 ESKD patients and 38 age- and sex-matched healthy controls. Diffusion tensor imaging (DTI) was performed using a three-tesla MRI scanner in patients with ESKD and healthy controls. We obtained PSMD based on DTI in several steps, including preprocessing, skeletonization, application of a custom mask, and histogram analysis. We compared PSMD between the groups and performed correlation analyses between PSMD and clinical factors.

**Results:**

PSMD was higher in patients with ESKD than in healthy controls (2.945 ± 0.361 vs. 2.552 ± 0.458 × 10^−4^ mm^2^/s, *p* < 0.001). In addition, PSMD positively correlated with age (*r* = 0.370, *p* = 0.022). In exploratory subgroup analyses, no statistically significant differences in PSMD were observed according to dialysis modality or the presence of diabetes mellitus.

**Conclusion:**

We demonstrated that patients with ESKD exhibited higher PSMD than healthy controls, suggesting diffuse white matter microstructural injury that may be related to CSVD. PSMD may be a useful imaging marker for characterizing diffuse white matter abnormalities in this population, although further studies incorporating conventional CSVD markers, vascular risk–matched controls, and clinical outcome measures are warranted.

## 1 Introduction

End-stage kidney disease (ESKD) represents the most advanced stage of chronic kidney disease and is associated with a substantially increased risk of cerebrovascular disease (1–4). This increased vulnerability may reflect the combined effects of impaired kidney function, vascular risk factors, endothelial dysfunction, and altered hemodynamic regulation. In patients receiving dialysis, additional hemodynamic and metabolic disturbances may further contribute to cerebral vascular injury (5, 6). Accordingly, increasing attention has been directed toward identifying subclinical cerebral abnormalities in patients with ESKD before overt neurological events occur.

Cerebral small vessel disease (CSVD) encompasses pathological processes affecting the brain’s small perforating arterioles, capillaries, and venules and is an important contributor to stroke, cognitive decline, and functional impairment (7–13). Kidney and brain microvasculature share several anatomical and hemodynamic characteristics, including exposure of small-caliber vessels to high pulsatile pressure and dependence on tightly regulated autoregulation (2, 14–16). This shared vulnerability underlies the concept of the cerebro-renal axis and may partly explain why impaired kidney function has been associated with silent lacunar infarction, white matter hyperintensities, and reduced white matter volume (15, 17–20). These observations suggest that patients with ESKD may be particularly susceptible to cerebral microvascular and white matter injury.

Conventional MRI markers of CSVD include white matter hyperintensities, lacunes, cerebral microbleeds, and enlarged perivascular spaces (7, 21). Although these markers are clinically useful, they may not fully capture diffuse microstructural abnormalities within the cerebral white matter, particularly at an early or subclinical stage. In addition, their assessment may depend on visual rating scales or lesion-based quantification, which can introduce observer-related variability and may incompletely reflect the overall burden of white matter injury (22–24). Accordingly, diffusion-based imaging measures have gained attention as potential complementary markers of diffuse white matter damage. Peak width of skeletonized mean diffusivity (PSMD) is an automated diffusion tensor imaging–derived measure that quantifies the dispersion of mean diffusivity values within the major white matter skeleton and has been shown to be sensitive to diffuse white matter microstructural injury associated with CSVD (25–27).

Despite growing evidence that impaired kidney function is associated with cerebral white matter abnormalities, PSMD has rarely been investigated specifically in patients with ESKD. Therefore, this study aimed to compare PSMD between patients with ESKD and age- and sex-matched healthy controls and to explore its associations with clinical characteristics. We hypothesized that patients with ESKD would exhibit higher PSMD, reflecting greater diffuse white matter microstructural abnormalities.

## 2 Methods

### 2.1 Participants

This prospective observational study was approved by the Institutional Review Board, and informed consent was obtained from all participants. We prospectively enrolled 38 patients with ESKD, each with a GFR of less than 15 mL/min/1.73m^2^, who were on dialysis for at least 3 months since they started dialysis. Patients with a history of neurological disease or structural brain lesions on MRI were excluded.

We also enrolled 38 age- and sex-matched healthy controls without a history of medical or neurological disease. Brain MRI in healthy controls also revealed no structural lesions.

Global cognitive function was assessed in patients with ESKD using the Korean version of the Montreal Cognitive Assessment (MoCA-K).

### 2.2 Diffusion tensor imaging scan

All DTI scans were performed using the same MRI scanner and imaging protocol in patients with ESKD and healthy controls. DTI was performed using a 3.0-T MRI scanner (AchievaTx, Phillips Healthcare, Best, The Netherlands) equipped with a 32-channel head coil.

DTI data were acquired using a spin-echo single-shot echo-planar imaging sequence with 32 diffusion directions (repetition time/echo time, 8,620/85 ms; flip angle, 90°; slice thickness = 2.25 mm, acquisition matrix, 120 × 120; field of view, 240 × 240 mm^2^; and *b*-value, 1,000 s/mm^2^).

### 2.3 Obtaining the PSMD

Figure 1 illustrates the process used to obtain PSMD from DTI data. Image processing was performed on a Linux platform using the FMRIB Software Library (FSL; Oxford Center for Functional MRI of the Brain, Oxford, United Kingdom). The process consists of four steps. The first step involved DTI preprocessing, including motion correction, eddy current correction, brain extraction, and tensor fitting. The second step involved skeletonization using tract-based spatial statistics, in which FA maps were registered to a common space and projected onto a mean white matter skeleton thresholded at an FA value of 0.2. The same transformation matrices were then applied to the mean diffusivity (MD) maps to generate skeletonized MD maps. The third step involved application of a custom mask to the skeletonized MD maps to exclude regions in which MD measurements may be less specific. The final step involved histogram analysis, in which PSMD was calculated as the difference between 95th and 5th percentiles of MD values from all voxels within the white matter skeleton.

**Figure 1:**
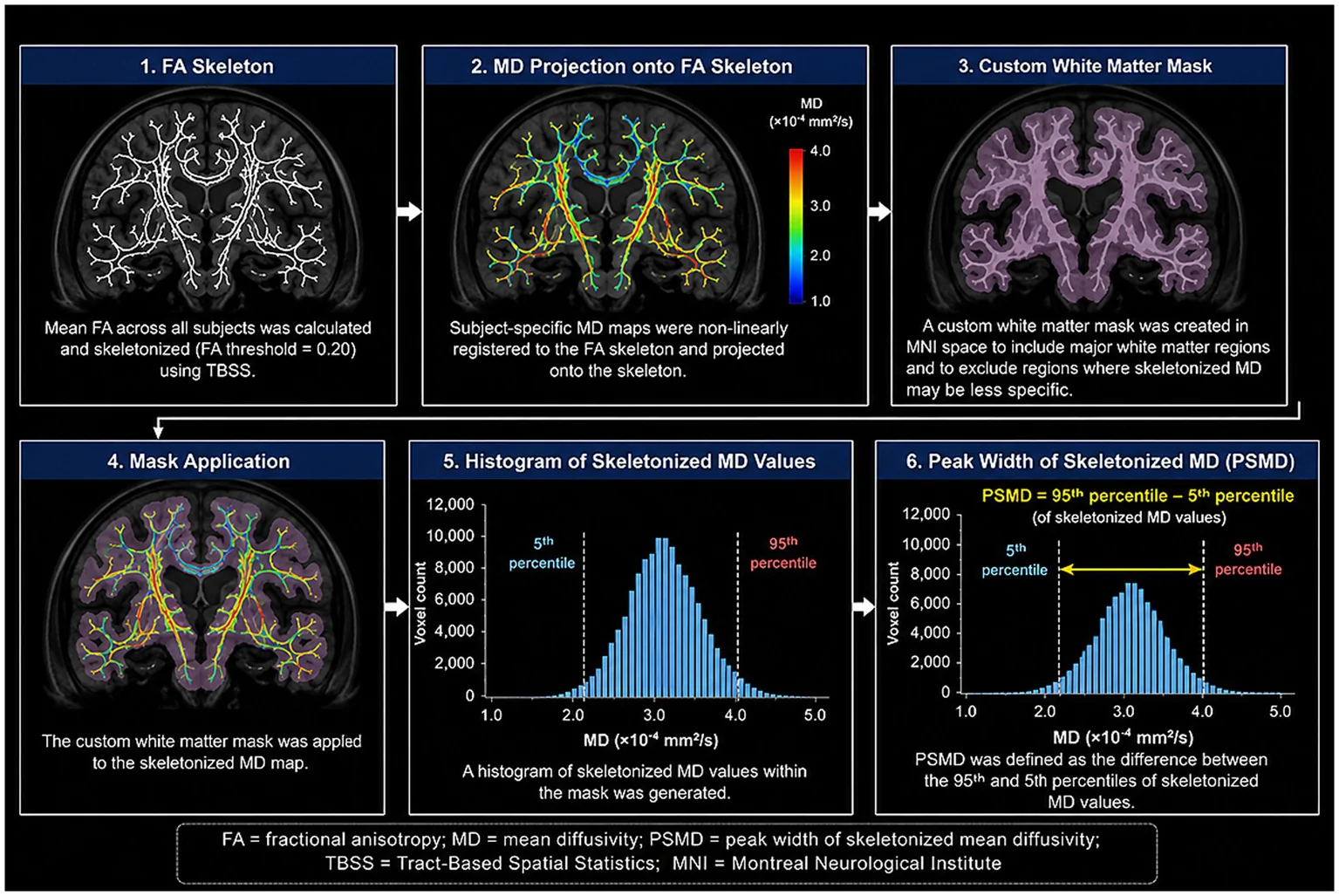
Workflow for calculation of peak width of skeletonized mean diffusivity (PSMD). Fractional anisotropy (FA) maps were aligned and skeletonized using tract-based spatial statistics (TBSS) to generate a white matter skeleton. Subject-specific mean diffusivity (MD) maps were then projected onto the FA skeleton. A custom white matter mask was applied to the skeletonized MD map to restrict the analysis to major white matter regions. A histogram of MD values within the masked skeleton was generated, and PSMD was calculated from the width of the MD distribution between the 95th and 5th percentiles. FA, fractional anisotropy; MD, mean diffusivity; PSMD, peak width of skeletonized mean diffusivity; TBSS, tract-based spatial statistics.

### 2.4 Statistical analysis

Continuous variables were expressed as mean ± standard deviation, and categorical variables were expressed as number and percentage. Independent-samples *t*-tests were used to compare age and PSMD values between patients with ESKD and healthy controls, and the chi-square test was used to compare sex distributions between the groups. Exploratory subgroup analyses using independent-samples *t*-tests were performed to compare PSMD according to dialysis modality (hemodialysis vs. peritoneal dialysis) and the presence or absence of diabetes mellitus. Pearson’s correlation analysis was used to explore associations between PSMD and clinical and laboratory variables, including the MoCA-K score, in patients with ESKD. Because the subgroup and correlation analyses were exploratory, correction for multiple comparisons was not applied, and these findings were interpreted cautiously. All tests were two-sided, and statistical significance was defined as *p* < 0.05. Statistical analyses were performed using MedCalc Statistical Software version 22.009 (MedCalc Software Ltd., Ostend, Belgium).

## 3 Results

### 3.1 Demographic and clinical characteristics of participants

Table 1 summarized the demographic and clinical characteristics of the patients with ESKD and healthy controls. Among the 38 patients with ESKD, 19 underwent hemodialysis and 19 underwent peritoneal dialysis. In addition, 20 patients had diabetes mellitus (DM), and 32 had hypertension. The mean MoCA-K score in patients with ESKD was 22.4 ± 5.3. Age and sex distributions did not differ significantly between patients with ESKD and healthy controls.

**Table 1:** Demographic and clinical characteristics of patients with ESKD and healthy controls.

Characteristics	Patients with ESKD (*N* = 38)	Healthy controls (*N* = 38)	*p*-value
Demographic data			
Age, years (SD)	61.7 (6.7)	61.5 (5.9)	0.928
Men, *N* (%)	17 (44.7)	14 (36.8)	0.486
Duration of dialysis, months (SD)	48.3 (49.9)		
Dialysis types (hemodialysis/peritoneal dialysis), *N* (%)	19 (50.0)/19 (50.0)		
Hypertension, *N* (%)	32 (84.2)		
Diabetes mellitus, *N* (%)	20 (52.6)		
Laboratory data			
Hemoglobin, g/dL (SD)	10.4 (1.1)		
Hematocrit, % (SD)	32.1 (3.5)		
Protein, g/dL (SD)	6.5 (0.5)		
Albumin, g/dL (SD)	3.7 (0.3)		
Aspartate aminotransferase, U/L (SD)	20.4 (7.3)		
Alanine aminotransferase, U/L (SD)	18.8 (9.8)		
Blood urea nitrogen, mg/dL (SD)	57.1 (16.9)		
Creatinine, mg/dL (SD)	8.9 (2.5)		
Sodium, mmol/L (SD)	139.0 (3.2)		
Potassium, mmol/L (SD)	4.6 (0.7)		
Chloride, mmol/L (SD)	99.6 (4.0)		
Calcium, mg/dL (SD)	8.4 (0.7)		
Phosphate, mg/dL (SD)	4.5 (0.9)		
Parathyroid hormone, pg./mL (SD)	273.7 (210.3)		
Total CO_2_ contents, mmol/L (SD)	24.7 (2.6)		
Cognitive function data			
MoCA-K score (SD)	22.4 (5.3)		

PSMD, peak width of skeletonized mean diffusivity; ESKD, end-stage kidney disease; SD, standard deviation; MoCA-K, Korean version of the Montreal Cognitive Assessment.

### 3.2 Difference in PSMD between the groups

PSMD was significantly higher in patients with ESKD than in healthy controls (2.945 ± 0.361 vs. 2.552 ± 0.458 × 10^−4^ mm^2^/s, *p* < 0.001) (Figure 2). In exploratory subgroup analyses, no statistically significant difference in PSMD was observed between patients undergoing hemodialysis and those undergoing peritoneal dialysis (2.905 ± 0.343 vs. 2.959 ± 0.398 × 10^−4^ mm^2^/s, *p* = 0.655). Similarly, PSMD did not differ significantly between patients with and without diabetes mellitus (2.926 ± 0.356 vs. 2.968 ± 0.376 × 10^−4^ mm^2^/s, *p* = 0.726).

**Figure 2:**
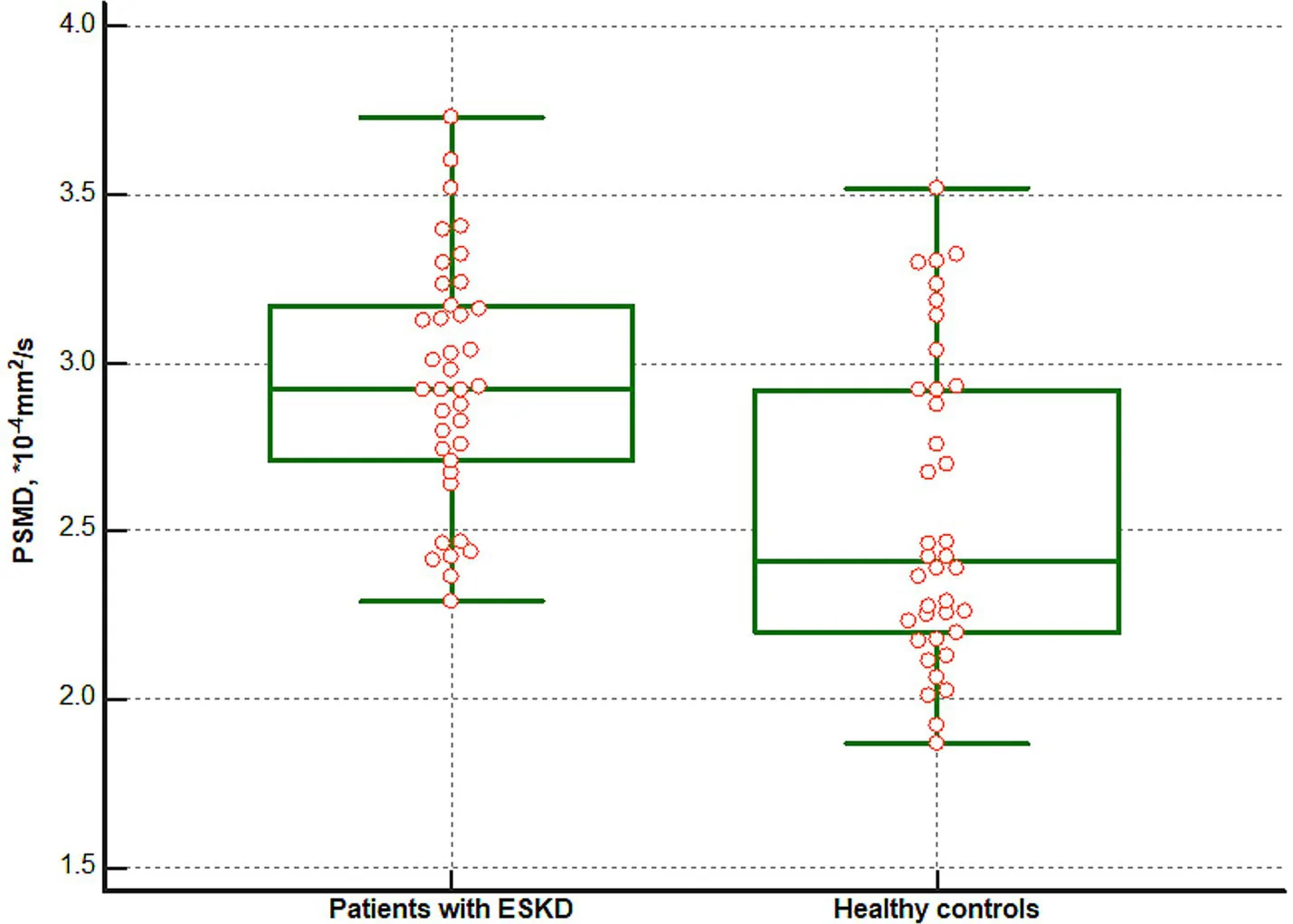
Comparison of peak width of skeletonized mean diffusivity (PSMD) between patients with end-stage kidney disease (ESKD) and healthy controls. PSMD was significantly higher in patients with ESKD than in healthy controls (2.945 ± 0.361 vs. 2.552 ± 0.458 × 10^−4^ mm^2^/s, *p < 0*.001). PSMD, peak width of skeletonized mean diffusivity; ESKD, end-stage kidney disease.

### 3.3 Correlation between the PSMD and clinical characteristics

In exploratory correlation analyses, PSMD was positively correlated with age in patients with ESKD (*r* = 0.370, *p* = 0.022) (Figure 3). No statistically significant correlations were observed between PSMD and the other evaluated clinical or laboratory variables, including dialyzer clearance time/distribution volume of urea (Kt/V) (*r* = −0.140, *p* = 0.396), duration of dialysis (*r* = −0.020, *p* = 0.904), albumin (*r* = −0.213, *p* = 0.193), total protein (*r* = −0.095, *p* = 0.565), blood urea nitrogen (*r* = 0.137, *p* = 0.404), creatinine (*r* = 0.308, *p* = 0.056), hemoglobin (*r* = −0.243, *p* = 0.136), hematocrit (*r* = −0.192, *p* = 0.242), aspartate aminotransferase (*r* = 0.001, *p* = 0.994), alanine aminotransferase (*r* = 0.148, *p* = 0.370), calcium (*r* = 0.075, *p* = 0.650), chloride (*r* = 0.084, *p* = 0.610), potassium (*r* = 0.156, *p* = 0.342), sodium (*r* = 0.023, *p* = 0.889), phosphate (*r* = 0.062, *p* = 0.706), and total CO₂ (*r* = −0.286, *p* = 0.077). PSMD was also not significantly correlated with the MoCA-K score (*r* = 0.053, *p* = 0.752).

**Figure 3:**
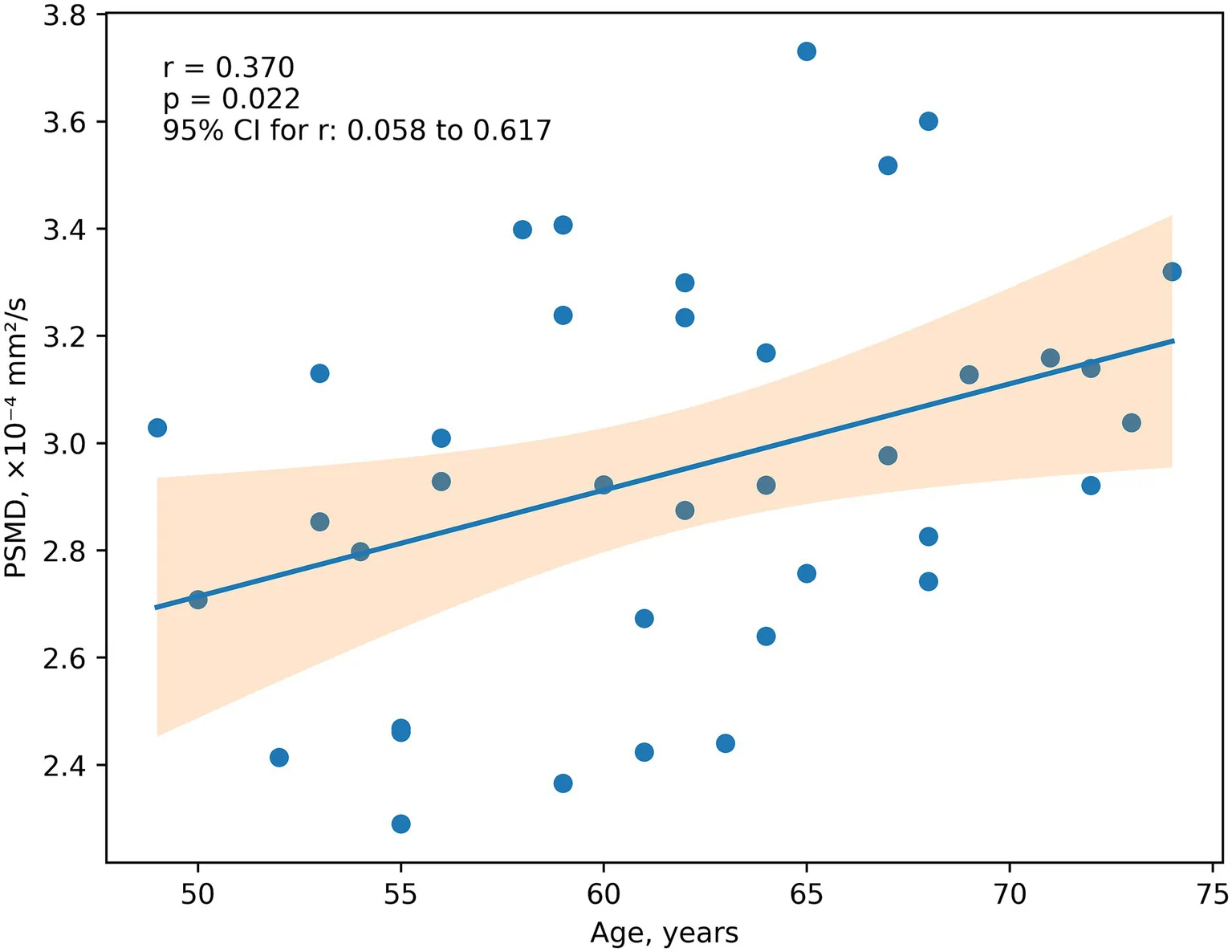
Correlation between age and peak width of skeletonized mean diffusivity (PSMD) in patients with end-stage kidney disease. PSMD was positively correlated with age in patients with ESKD (Pearson’s *r = 0*.370, *p = 0*.022; 95% *CI*, 0.058–0.617). The solid line represents the fitted linear regression line, and the shaded area represents the 95% confidence interval. PSMD, peak width of skeletonized mean diffusivity; ESKD, end-stage kidney disease.

## 4 Discussion

In this study, patients with ESKD exhibited significantly higher PSMD than age- and sex-matched healthy controls, indicating greater diffuse white matter microstructural abnormalities. In exploratory analyses, PSMD was positively associated with age, whereas no statistically significant differences were observed according to dialysis modality or the presence of diabetes mellitus. These findings suggest that PSMD may be useful for characterizing diffuse white matter injury in patients with ESKD. However, elevated PSMD should not be interpreted as direct evidence of CSVD because conventional CSVD imaging markers were not systematically assessed in this study. Moreover, because hypertension and diabetes mellitus were substantially more prevalent in the ESKD group than in the healthy control group, the observed difference in PSMD may reflect the combined effects of advanced kidney disease and associated vascular and metabolic comorbidities rather than an independent effect of ESKD alone.

Several mechanisms may contribute to diffuse white matter microstructural injury in patients with ESKD. Chronic hypertension, endothelial dysfunction, oxidative stress, inflammation, and impaired vascular autoregulation are common in advanced kidney disease and may promote cerebral hypoperfusion, blood–brain barrier dysfunction, and microvascular injury (2, 14, 19, 21). Because the cerebral small vessels and renal microvasculature share similar anatomical and hemodynamic characteristics, these systemic vascular abnormalities may preferentially affect vulnerable small vessels within the brain (2, 14, 19, 21). Previous studies have also reported associations between impaired kidney function and conventional neuroimaging manifestations of cerebral small vessel injury, including white matter hyperintensities, lacunar infarction, and cerebral microbleeds (28, 29). Hypertension, a common comorbidity in ESKD, is closely linked to activation of the renin–angiotensin system and may contribute to endothelial dysfunction within the cerebral microvasculature (30). Oxidative stress and autonomic dysfunction may further promote blood–brain barrier disruption and vascular injury (31). Consistent with these widespread vascular and metabolic disturbances, cognitive impairment is more common in patients with CKD than in the general population (32). Taken together, these mechanisms provide a plausible biological framework for the greater diffuse white matter microstructural abnormalities observed in the present ESKD cohort, although the current study cannot determine the relative contributions of ESKD itself and its accompanying vascular comorbidities.

PSMD is a diffusion-derived measure that reflects the dispersion of mean diffusivity values within the major white matter skeleton and has been proposed as a sensitive marker of diffuse white matter microstructural damage (25). Previous studies have demonstrated that elevated PSMD is associated with greater CSVD burden and cognitive impairment, supporting its potential role as a complementary marker of diffuse cerebral white matter injury (25). These findings are also consistent with the cerebro–renal axis hypothesis, which highlights the shared vulnerability of cerebral and renal microvasculature to systemic vascular injury (33). Furthermore, glymphatic system dysfunction has been reported in patients with ESKD, suggesting that disturbances in perivascular fluid transport may coexist with other forms of cerebral microvascular and white matter injury (34). However, whether such alterations are directly related to elevated PSMD remains unknown. In the present study, the higher PSMD observed in patients with ESKD supports the presence of greater diffuse white matter microstructural abnormalities compared with healthy controls. Importantly, because PSMD is not specific to CSVD and conventional CSVD imaging markers were not systematically assessed, our findings should not be interpreted as direct evidence of an increased CSVD burden.

Because hemodialysis and peritoneal dialysis differ substantially in their hemodynamic and inflammatory profiles, we explored whether PSMD varied according to dialysis modality. Hemodialysis is associated with intermittent hemodynamic fluctuations that may influence cerebral perfusion and potentially contribute to white matter injury (35). In contrast, previous studies have reported a higher burden of conventional CSVD-related imaging abnormalities, including lacunar infarcts, cerebral microbleeds, and white matter hyperintensities, in some patients undergoing peritoneal dialysis (36). Chronic inflammation related to repeated peritoneal exposure and dialysis-related factors has been proposed as one possible mechanism underlying these findings (37). In the present study, however, no statistically significant difference in PSMD was observed between hemodialysis and peritoneal dialysis. This exploratory finding should be interpreted cautiously because each subgroup included only 19 patients and was therefore likely underpowered to detect modest between-group differences. In addition, PSMD reflects diffuse white matter microstructural heterogeneity and may capture a pathological dimension that differs from conventional lesion-based CSVD markers. Accordingly, the present findings do not establish equivalence between the two dialysis modalities or exclude modality-specific effects on cerebral white matter injury.

Diabetes mellitus is a major cause of ESKD and was present in more than half of the patients in our cohort (38). Previous studies have consistently shown that type 2 diabetes is associated with an increased risk of cognitive impairment and dementia, as well as a greater burden of white matter hyperintensities and other cerebral microvascular abnormalities (39–43). We therefore explored whether PSMD differed according to the presence of diabetes mellitus. In the present study, no statistically significant difference in PSMD was observed between patients with and without diabetes. However, this exploratory subgroup analysis was based on a relatively small sample and should not be interpreted as evidence that diabetes has no additional effect on white matter microstructural injury. Moreover, because all participants had advanced ESKD with a substantial burden of vascular and metabolic abnormalities, any additional contribution of diabetes may have been difficult to detect. Larger studies with adequate statistical power and adjustment for vascular risk factors are needed to clarify the independent association between diabetes and PSMD in patients with ESKD.

We also examined the relationship between PSMD and global cognitive function assessed using the MoCA-K but found no significant association. Although previous studies have linked elevated PSMD to poorer cognitive performance, the absence of a significant association in our cohort may reflect the relatively small sample size, heterogeneity of cognitive performance, or the limited sensitivity of a global screening measure to specific cognitive domains affected by diffuse white matter injury. This exploratory finding should therefore be interpreted cautiously, and future studies incorporating comprehensive domain-specific neuropsychological assessments are warranted.

We also observed a positive correlation between age and PSMD in patients with ESKD. This finding is consistent with previous work demonstrating that PSMD increases across the adult lifespan and may reflect age-related alterations in white matter microstructure (44). However, the present correlation analysis was exploratory and was not corrected for multiple comparisons. Therefore, the association between age and PSMD should be interpreted cautiously and considered hypothesis-generating rather than confirmatory. Nevertheless, the observed relationship suggests that age may contribute to the heterogeneity of white matter microstructural injury in patients with ESKD and should be carefully considered in future studies evaluating PSMD in this population.

Several limitations should be considered when interpreting the present findings. First, this was a single-center study with a relatively small sample size, which may limit the generalizability of the results and reduce statistical power, particularly in the exploratory subgroup analyses. Second, the ESKD group had a substantially higher burden of vascular and metabolic comorbidities, including hypertension and diabetes mellitus, than the healthy control group. Because these factors may independently affect cerebral white matter microstructure, the observed between-group difference in PSMD cannot be attributed to ESKD alone. A vascular risk–matched non-ESKD control group or adequately powered multivariable analyses would be needed to clarify the independent contribution of ESKD. Third, conventional MRI markers of CSVD, including white matter hyperintensities, lacunes, cerebral microbleeds, and enlarged perivascular spaces, were not systematically assessed. Although global cognitive function was assessed using the MoCA-K, comprehensive domain-specific neuropsychological assessments were not incorporated into the present analysis. Therefore, the relationship between PSMD, conventional CSVD burden, and specific cognitive domains could not be fully determined. Fourth, MRI timing relative to hemodialysis sessions was not standardized. Acute hemodynamic and osmotic changes associated with hemodialysis may influence diffusion measurements, and sensitivity analyses according to scan timing were not feasible in the present dataset. Fifth, the cross-sectional design precludes causal inference regarding the relationship between ESKD and white matter microstructural abnormalities. Finally, the subgroup and correlation analyses were exploratory and were not corrected for multiple comparisons; therefore, particularly the observed association between age and PSMD should be interpreted cautiously and requires confirmation in larger independent cohorts.

## 5 Conclusion

Patients with ESKD exhibited higher PSMD than age- and sex-matched healthy controls, indicating greater diffuse white matter microstructural abnormalities. These findings suggest that PSMD may serve as a useful imaging marker for characterizing diffuse white matter injury in this population. However, because PSMD is not specific to CSVD and vascular and metabolic comorbidities were more prevalent in the ESKD group, the observed differences should not be interpreted as direct evidence of CSVD or as an independent effect of ESKD. Further studies incorporating conventional CSVD imaging markers, vascular risk–matched controls, comprehensive domain-specific cognitive assessments, and standardized MRI timing are warranted.

## Data Availability

The raw data supporting the conclusions of this article will be made available by the authors, without undue reservation.
